# Serial evaluation of cardiac biomarker NT-proBNP with speckle tracking echocardiography in a 6-year-old Golden Retriever dog with subaortic stenosis and dilated cardiomyopathy

**DOI:** 10.1080/01652176.2020.1727992

**Published:** 2020-02-25

**Authors:** Woong-Bin Ro, Min-Hee Kang, Hee-Myung Park

**Affiliations:** Department of Veterinary Internal Medicine, College of Veterinary Medicine, Konkuk University, Seoul, Republic of Korea

**Keywords:** Dog, canine, dilated cardiomyopathy, DCM, subaortic stenosis, NT-proBNP, speckle tracking echocardiography

A 6-year-old, 37.5-kg, castrated male Golden Retriever was presented with intermittent coughing and cardiac murmur. Physical examination revealed a grade III/VI systolic heart murmur at the mitral valve area, tachycardia, and a resting respiratory rate (RRR) of 24 breaths/min. Blood pressure was within normal range (systolic pressure 129 mmHg, diastolic pressure 104 mmHg, and mean arterial pressure 112 mmHg [Cardell 9402 Veterinary Monitor; Midmark, Tampa, FL, USA]). Complete blood count and serum chemistry results were within normal range. Thoracic radiography demonstrated left-sided cardiomegaly and a moderate broncho-interstitial pattern in the perihilar region. The electrocardiography (ECG) revealed a mean electrical axis of 86˚, sinus tachycardia (168 bpm), a widened P wave (0.06 s, [reference value, ≤0.05 s]) (Martin 2015), increased R amplitude (5.2 mV, [reference value, 2.61 ± 0.55 mV]) (Sato et al. 2000), and depressed ST segment in lead II.

**Figure 1.**
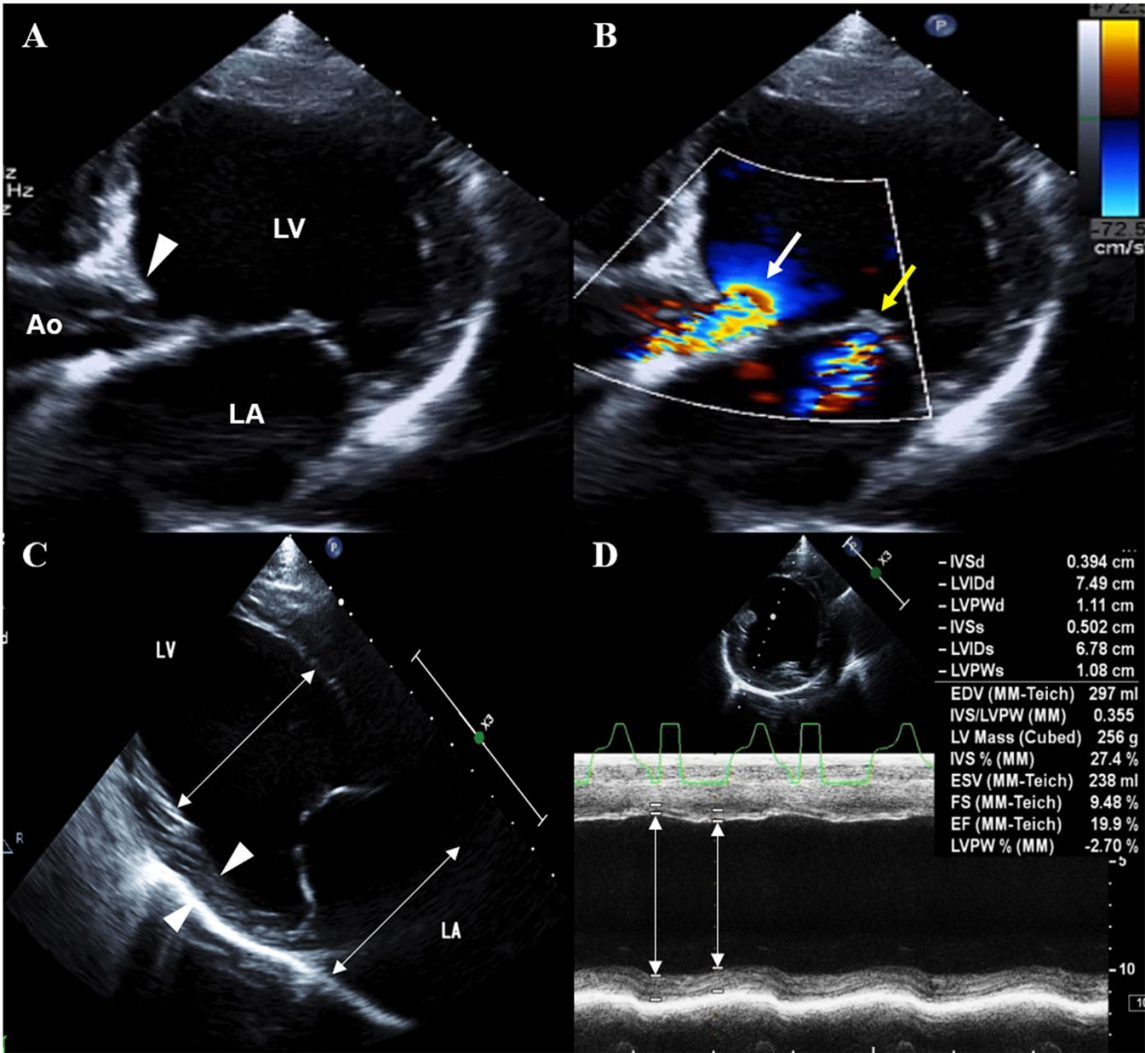
(A) B-mode and (B) color-flow Doppler echocardiography of the left apical five-chamber view in a dog with SAS and DCM showing class-2 SAS characterized by a linear fibrous structure on LVOT (arrow head) and turbulent subvalvular systolic flow (white arrow). Mitral regurgitation was also observed (yellow arrow). (C) Two-dimensional echocardiogram (right parasternal long-axis view) showing DCM characterized by dilated chambers. Note the enlargement of LV and LA (double arrowed lines) and the thin walls of the ventricle (arrow heads). (D) Poor systolic function (fractional shortening 9.48%) documented on M-mode (right parasternal short-axis view). Note the lack of contractility (double arrowed lines). Ao, Aorta; DCM, dilated cardiomyopathy; LA, left atrium; LV, left ventricle; LVOT, left ventricular outflow tract; SAS, subaortic stenosis.

On two-dimensional (2D) echocardiography (EPIQ 7 ultrasound system, Philips Medical Systems, Andover, MA, USA), a class-2 subaortic stenosis (SAS) was identified, characterized by a linear fibrous structure on the left ventricular outflow tract and severe post-stenotic dilation of the aorta (Figure 1A). Color flow Doppler imaging of the left ventricular outflow tract showed turbulent subvalvular systolic flow (Figure 1B). An increased flow velocity across the left ventricular outflow tract was measured by continuous-wave Doppler (4.34 m/s, [reference value, 1.29 ± 0.22 m/s], pressure gradient 75 mmHg) (Chetboul et al. 2005), and aortic regurgitation was documented by color Doppler and continuous-wave Doppler (3.07 m/s; pressure gradient 38 mmHg). Furthermore, a dilated cardiomyopathy (DCM) was diagnosed which is characterized by a dilated left ventricle (LV) and left atrium {E point to septal separation 31 mm, [reference value, 1–10 mm], LV internal diameter in diastole (LVIDd) 74.9 mm, LVIDd indexed to body weight 2.58, [reference value, 1.35-1.73], LV internal diameter in systole (LVIDs) 67.8 mm, LVIDs indexed to body weight 2.16, [reference value, 0.79–1.14], end diastolic volume index (EDVI) 258 mL/m^2^, [reference value, 54.16 ± 1.86 mL/m^2^], left atrial to aortic root dimension ratio 2.6, [reference value, 1.23 ± 0.03]} (Figure 1C), with poor systolic function {end systolic volume index (ESVI) 207 mL/m^2^, [reference value, 21.53 ± 1.15 mL/m^2^], fractional shortening (FS) 9.48%, [reference value, 27–55%], ejection fraction (EF) in Simpson’s method 8.0%, [reference value, 61.00 ± 1.24%]} (Morrison et al. 1992; Saini et al. 2017; Cornell et al. 2004) (Figure 1D). The sphericity index of LV was 1.15 (<1.65 represents increased sphericity) (Dukes-McEwan et al. 2003). A central jet of mitral regurgitation was documented by color Doppler and continuous-wave Doppler.

The dog was administered pimobendan (0.3 mg/kg BW, PO q 12 h), furosemide (1 mg/kg BW, PO q 12 h), ramipril (0.125 mg/kg BW, PO q 24 h), diltiazem sustained-release (3 mg/kg BW PO, q 12 h), spironolactone (1 mg/kg BW PO, q 12 h), and digoxin (0.005 mg/kg BW PO, q 12 h). To minimize the risk of myocardial damage, taurine (1 g/dog, PO q 12 h) and L-carnitine (2 g/dog, PO q 12 h) were supplemented to the cardiac medication described above. The dog’s owner refused to perform interventional approach to SAS, such as balloon valvuloplasty.

**Figure 2.**
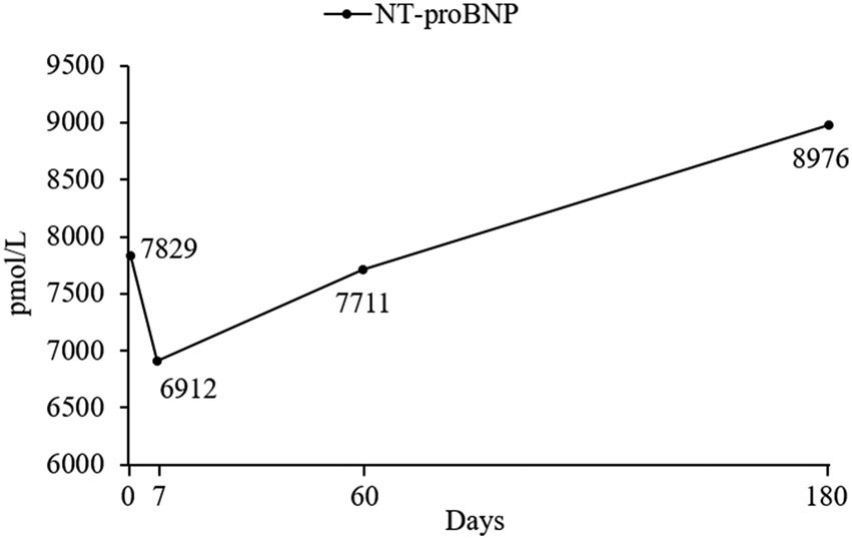
Serial evaluation of serum NT-proBNP concentration in a dog with SAS and DCM. DCM, dilated cardiomyopathy; NT-proBNP, N-terminal pro B-type natriuretic peptide; SAS, subaortic stenosis.

**Figure 3.**
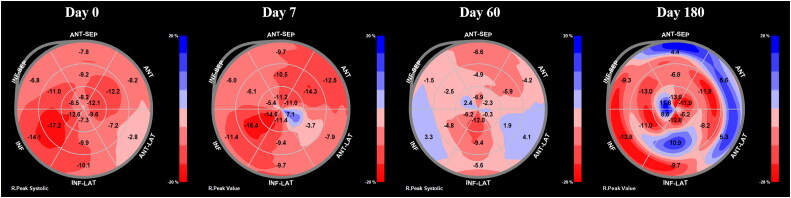
Serial evaluation of bull’s eye map of longitudinal strain by two-dimensional speckle tracking echocardiography performed in a dog with SAS and DCM. On day 7, segmental dyskinesia (blue region) in the apical region was detected, even though the overall results in radial and circumferential evaluations were improved after treatment. This deterioration of cardiac function in a specific segment was undetectable with conventional echocardiography and serum NT-proBNP evaluation. The segmental dyskinesia gradually worsened with disease progression. DCM, dilated cardiomyopathy; NT-proBNP, N-terminal pro B-type natriuretic peptide; SAS, subaortic stenosis.

The serum concentration of N-terminal pro B-type natriuretic peptide (NT-proBNP) (IDEXX Laboratories Inc., Westbrook, ME, USA), conventional echocardiography, and 2D speckle tracking echocardiography (2D-STE) were serially evaluated in addition to other clinical examinations {heart rate (HR), heart rhythm on ECG, and RRR} on days 0, 7, 60, and 180. The serial changes of serum NT-proBNP concentration, bull’s eye map by 2D-STE, and echocardiographic results are shown in Figures 2 and 3, and Table 1, respectively. The images for 2D-STE analysis were obtained and analyzed using a QLAB 7.0 system (cardiac motion quantification; Philips Medical Systems, Best, the Netherlands). 2D cine loops of parasternal or apical view with three or more consecutive cardiac cycles and high frame rates (40-60 frames/s) were stored digitally for subsequent offline analysis. LV myocardium was sufficiently visualized by careful acquisition of 2D views. The software automatically selected a starting reference image which began with the R-wave of the electrocardiogram. Then, the bilateral annuli and the apex of the LV myocardial wall were manually selected by the investigator. The endocardial border of the myocardium was automatically traced by the software, designating a region of interest (ROI). If necessary, the investigator manually corrected the ROI to incorporate the entire endocardial surface without any artifacts. The investigator ensured that myocardial movement was visually synchronized with the ROI for the entire cardiac cycle. The ROI was divided into six segments and the segmental values were averaged to obtain mean LV strain and strain rate. The velocity, strain and strain rate values in this case were the peak values on the respective mean curves. The LV radial velocity (RV) and radial FS (RFS) were analyzed by 2D-STE from standard right-parasternal short-axis view at the mid-ventricular level (papillary muscle level). The LV circumferential strain (CS) and circumferential strain rate were evaluated from the same view used in the radial evaluation. The LV longitudinal strain (LS) and longitudinal strain rate were evaluated from three different views (standard left-apical four-chamber, three-chamber, and two-chamber views), and the average was calculated for global LS and longitudinal strain rate. A bull’s eye map was created for complete analysis of the LV longitudinal function.

**Table 1.** Serial evaluation of conventional echocardiography and 2D-STE in a 6-year-old Golden Retriever dog with subaortic stenosis and dilated cardiomyopathy.

Variable	Day 0	Day 7	Day 60	Day 180
Heart rate (bpm)	168	134	131	132
**Conventional echocardiography**				
FS (%)	9.48	12.7	16.7	13.5
EF (%)	8.0	14.5	19.5	16.4
EDVI	258	240	252	253
ESVI	207	177	173	173
**2D-STE**				
Radial				
Velocity (cm/s)	3.4	4.5	5.4	4.0
FS (%)	7.3	8.2	9.7	7.5
Circumferential				
St (%)	−7.2	−8.2	−9.6	−7.5
SRS (1/s)	−1.0	−1.5	−1.6	−1.4
Global longitudinal				
St (%)	−9.6	−9.7	−8.0	−5.4
SRS (1/s)	−1.7	−1.7	−1.4	−1.2

EDVI, end diastolic volume index; EF, ejection fraction in Simpson’s method; ESVI, end systolic volume index; FS, fractional shortening; SRS, strain rate in systole; St, strain.

On day 0, the serum NT-proBNP concentration was 7829 pmol/L [reference value, 0 to 900 pmol/L] (Figure 2). The 2D-STE results showed a clear reduction in the CS (−7.2%, [reference value, −25 to −19%]), circumferential strain rate in systole (CSRS) (−1.0/s, [reference value, −2.9 to −2.3/s]), global LS (−9.6%, [reference value, −23 to −14%]), and global longitudinal strain rate in systole (LSRS) (−1.7/s, [reference value, −2.8 to −1.9/s]) (Suzuki et al. 2013) (Table 1).

On day 7, the clinical sign of intermittent coughing disappeared and the clinical examination showed RRR of 18 breaths/min and sinus rhythm (134 bpm). The serum NT-proBNP concentration decreased to 6912 pmol/L [reference value, 0 to 900 pmol/L] after treatment (Figure 2). In conventional echocardiography and 2D-STE evaluation, FS, EF, EDVI, ESVI, RV, RFS, CS, and CSRS were improved, and global LS and LSRS remained similar to day 0 (Table 1). However, segmental dyskinesia in apical region was detected on a bull’s eye map of LS (Figure 3).

On day 60, the dog showed RRR of 24 breaths/min and sinus rhythm (131 bpm). The serum NT-proBNP concentration increased to 7711 pmol/L [reference value, 0 to 900 pmol/L], indicating worsened myocardial damage (Figure 2). The conventional echocardiography and 2D-STE results showed improvements in FS, EF, RV, RFS, CS, CSRS, and ESVI, but deteriorations in EDVI, global LS, and global LSRS compared to day 7 (Table 1). The bull’s eye map of LS demonstrated progression of myocardial dyskinesia (Figure 3).

On day 180, the dog showed RRR of 24 breaths/min and sinus rhythm (132 bpm). The serum NT-proBNP concentration increased continuously to 8976 pmol/L [reference value, 0 to 900 pmol/L] (Figure 2). The FS, EF, RV, RFS, CS, CSRS, global LS, and global LSRS worsened, while EDVI and ESVI remained similar to day 60 (Table 1). The bull’s eye map of LS identified even more intensified myocardial dyskinesia than that on day 60 (Figure 3).

On day 229, the dog exhibited vomiting, hyporexia, and severe tachycardia (240 bpm). Electrocardiography revealed atrial fibrillation with notched R wave. Without response to esmolol (constant rate infusion of 0.1–0.2 mg/kg BW/min) and sotalol (2 mg/kg BW PO, q 12 h), the atrial fibrillation persisted and the dog died the following day.

Subaortic stenosis (SAS) is one of the most common congenital cardiac diseases in dogs (Kleman et al. 2012), which results in LV concentric hypertrophy by pressure overload (O’Grady et al. 1989). Dilated cardiomyopathy (DCM) is also one of the most common cardiomyopathies in dogs (Borgarelli et al. 2001), characterized by LV dilation, impaired systolic function, and hemodynamic state of volume overload (Dukes-McEwan et al. 2003; Tidholm et al. 2001). However, concurrence of the two diseases has been rarely reported in dogs. There is one retrospective study reporting two dogs with SAS and concurrent DCM among 195 dogs with confirmed diagnosis of SAS, but no detailed description and evaluation of cardiac function and disease progression were made (Kienle et al. 1994). To the authors’ knowledge, this is the first case report describing a serial change of cardiac function and myocardial damage during monitoring period in a dog with SAS and DCM, which is a rare condition with two different hemodynamic properties.

The dog in this case mainly exhibited characteristics of DCM rather than those of SAS. However, due to concomitant SAS, myocardial dysfunction and cardiac remodeling of the dog were clearly worse than those of the previously reported DCM dogs (Pedro et al. 2017; Bélanger et al. 2005). In addition, the median survival time of pimobendan-treated DCM dogs were 1037 days in five Cocker Spaniels and 329 days in five Doberman Pinschers (Fuentes et al. 2002), whereas the dog in this case with DCM and SAS died much earlier.

In veterinary medicine, it is known that SAS can develop to show LV dilation and reduced EF in end-stage (De Madron et al. 2016), similar to the “low-flow, low gradient (LF-LG) aortic stenosis (AS)” in humans (Pibarot and Dumesnil 2012). However, no case of SAS has been reported in dogs that have caused myocardial changes to meet the diagnostic criteria of canine DCM (Tidholm et al. 2001), and the exact diagnostic criteria or characteristic features of LF-LG AS are not well demonstrated in dogs. The dog in the present case showed clear myocardial dysfunction and cardiac remodeling which fulfilled all proposed major and minor criteria for diagnosis of canine DCM (Dukes-McEwan et al. 2003). Nevertheless, since the histopathology of this case was not available, there is also a possibility of LF-LG AS, like in humans. Therefore, “LF-LG AS-like disease” should be considered as a differential diagnosis of the dog in this case. Further studies on end-stage SAS and establishment of criteria for LF-LG AS are required in dogs.

For a precise assessment of cardiac function and identification of myocardial damage, serum NT-proBNP concentration and 2D-STE were used to monitor the dog of the present case over time. The NT-proBNP is considered to be one of the most specific and widely used cardiac biomarkers in dogs, especially in volume overload conditions where stress or stretch of the myocardium occurs (Oyama 2015; Wess et al. 2011; Singletary et al. 2012). Nevertheless, combination of NT-proBNP with other methods are required for detection of early cardiac disease (Oyama 2015; Singletary et al. 2012). In human heart diseases, the 2D-STE has been evaluated in combination with NT-proBNP and showed significant correlation with NT-proBNP (Mornos et al. 2011; Wang et al. 2012; Meimoun et al. 2011). However, in veterinary medicine, there are no reports comparing the changes of NT-proBNP and 2D-STE in heart diseases during treatment period. This case delineates how 2D-STE can be applied in combination with NT-proBNP to monitor a dog with myocardial injury.

The most notable point of this case is that the 2D-STE measurement was able to detect cardiac dysfunction in a specific segment which was undetectable by clinical examinations and NT-proBNP measurement. On day 7, clinical signs, HR, LV contractility and myocardial damage were improved after treatment, which were confirmed by the results of clinical examinations, conventional echocardiography, 2D-STE, and NT-proBNP. However, despite the improvement of overall myocardial function, a segmental dyskinesia in apical segment was detected in bull’s eye map of LS. This suggests that improvement of the overall myocardial injury does not mean improvement of all myocardial segments, and some of the myocardial segments may be exacerbated in spite of increased contractility. Therefore, the combined use of biomarkers such as NT-proBNP with 2D-STE in dogs with myocardial damage may be useful for accurate assessment of myocardial status.

Regarding the response to therapy on day 7, the possibility of tachycardia induced cardiomyopathy (TIC) as a primary cause of DCM can arise because clinical signs and myocardial function were improved simultaneously with resolution of tachycardia in response to the therapy (Shinbane et al. 1997; Zupan et al. 1996). It is known that TIC can mimic DCM on echocardiography and is characterized to be largely reversible by resolution of tachycardia (Martin 2015, p. 102–111, Shinbane et al. 1997; Zupan et al. 1996). However, the tachycardia might have been secondary to the primary cardiac disorders since the dog showed sinus tachycardia with evidence of congestive heart failure (CHF), rather than supraventricular or ventricular tachycardia (Martin 2015). In addition, the results of global LS and NT-proBNP concentration deteriorated even though the HR remained stable at day 60 and 180, which also indicates that the tachycardia was not the primary cause of the myocardial dysfunction (Packer et al. 1986). The improvement of clinical signs and myocardial function on day 7 could be due to improvement of secondary TIC (resolution of tachycardia), however the improvements including the reduction of HR might also be due to resolution of CHF and increased cardiac output by cardiac medications. Therefore, TIC was not considered as a primary cause of DCM, although TIC secondary to primary cardiac disorders could be a possible differential diagnosis in this dog.

Of the various 2D-STE indices used in this case, the longitudinal deformations (global LS and global LSRS) were the most sensitive and accurate indicators of the myocardial damage detected by NT-proBNP. From day 7 to day 180, the values of longitudinal deformations decreased as serum NT-proBNP concentration increased. The high sensitivity of the longitudinal deformations to myocardial damage has also been reported in human studies (Mizuguchi et al. 2008; Kouzu et al. 2011). The three-directional 2D-STE results in patients with cardiovascular risk factors and diastolic dysfunction showed lower LS and LSRS than those of control patients (Mizuguchi et al. 2008). In another study on three-directional 2D-STE in hypertension patients with eccentric cardiac hypertrophy, a significant decrease was observed only in longitudinal deformation (Kouzu et al. 2011). The longitudinal fibers of the heart consist mainly of subendocardial fibers, which are the most vulnerable fibers to interstitial fibrosis and ischemia, and therefore, it is known that the longitudinal function is most sensitive to the initial changes of cardiac disease (Kouzu et al. 2011). Thus, also in veterinary medicine, the longitudinal deformations have possibility to be the most sensitive and useful indicator of myocardial damage, especially in dogs with DCM. However, in the prior study on 2D-STE in DCM dogs (Pedro et al. 2017), longitudinal deformations were not measured. Therefore, further investigation on the directional difference of 2D-STE in DCM dogs is required.

Meanwhile, radial and circumferential deformations (RV, RFS, CS, and CSRS) were the most sensitive indicators of myocardial contractility. Both radial and circumferential deformations, as well as FS and EF increased on day 7 and day 60. The increase on day 7 is thought to be a response to pimobendan administration, and the increase on day 60 may be a compensation for the decrease in longitudinal deformation. In both humans and dogs (Suzuki et al. 2013; Mizuguchi et al. 2008; Kouzu et al. 2011), it is reported that the myocardial contractility is mainly exerted on radial and circumferential directions, and the radial and circumferential deformations increase in compensation for longitudinal dysfunction. This suggests that radial and circumferential deformations in 2D-STE can be used as an accurate indicator of contractility in DCM dogs. However, as observed on day 60, an increase in contractility does not indicate improvement of the myocardium. Therefore, for precise assessment of myocardial function in dogs with myocardial damage, monitoring using all three directions of 2D-STE will be necessary.

In this case, segmental dyskinesia on day 7 occurred initially in the apical region. It is reported in human DCM patients that myocardial shortening in the apex precedes that in the middle and basal regions, and thus the apical region was affected most by the wall stress (Fujita et al. 1993). Therefore, also in dogs, the apical region may be the first site of injury in cardiomyopathies such as DCM. Additional retrospective studies in large number of dogs with cardiomyopathies are expected to clarify this.

In conclusion, this is the first reported case of a dog with DCM and SAS, which described and compared serial changes of NT-proBNP and 2D-STE with disease progression. The 2D-STE was able to detect segmental dyskinesia which was undetectable by NT-proBNP measurement. In addition, longitudinal deformations were the most sensitive 2D-STE indices to show the myocardial damage, while radial and circumferential deformations were accurate indices to show myocardial contractility. Therefore, monitoring with NT-proBNP and 2D-STE is expected to have great diagnostic and prognostic utility in dogs with heart diseases. Further large-scale studies on the comparison and clinical application of cardiac biomarkers with 2D-STE are recommended.
